# Is the AKIN score useful as an indicator of the optimum time for intervention with renal replacement therapy in critically ill patients?

**DOI:** 10.1186/cc10967

**Published:** 2012-03-20

**Authors:** S Mousdale, J Bannard-Smith

**Affiliations:** 1 Royal Blackburn Hospital, Blackburn, UK

## Introduction

Acute kidney injury represents a significant workload and economic burden for critical care units. In the critical care setting AKI is usually associated with a variety of aetiologies such as septic shock, major surgery and heart failure [1]. Controversy exists as to the optimal time for the institution of renal replacement therapy (RRT) [2]. Scoring systems such as AKIN have used the rise in serum creatinine combined with reduced urine output over a period of 48 hours as indicative of the degree of injury [3]. We used this scoring system to see if ITU mortality correlated with increasing AKIN score.

## Methods

The Critical Care Minimum Dataset records of patients admitted to our mixed general ICU were investigated. Those patients who received renal organ support were investigated further. The change in serum creatinine levels in the 48 hours prior to institution of RRT was used to determine the AKIN score. Patients in whom there was not a significant rise in creatinine, but who received RRT, were staged zero. Unfortunately, urine output data were not available to improve accuracy.

## Results

There were a total of 276 patients whose records were adequate for this audit. Several records were incomplete and not used. Demography and APACHE II scores were similar across all groups. Length of stay and days of RRT were similar across the groups. ICU survival was as follows: AKIN stage: (0) 42.2%, (1) 50.6%, (2) 51.7%, (3) 70.4%. Pearson chi-square *P *< 0.001.

## Conclusion

We were not able to demonstrate improved survival when RRT was initiated at an earlier AKIN stage. A small nonsignificant trend was observed with increasing stage and the differences between groups were significant. Very early initiation of RRT was associated with increased mortality. Stage (3) included patients with chronic kidney disease, which probably skewed the results in this group. We cannot recommend the use of the AKIN score as a pointer to when to initiate RRT, based on these data.
